# DNA Logic Gate Based on Metallo-Toehold Strand Displacement

**DOI:** 10.1371/journal.pone.0111650

**Published:** 2014-11-03

**Authors:** Wei Deng, Huaguo Xu, Wei Ding, Haojun Liang

**Affiliations:** 1 CAS Key Laboratory of Soft Matter Chemistry, Department of polymer science and engineering, University of Science and Technologyof China, Hefei, Anhui, P. R. China; 2 Hefei National Laboratory for Physical Sciences at the Microscale, University of Science and Technology of China, Hefei, Anhui, P. R. China; Northwestern University Feinberg School of Medicine, United States of America

## Abstract

DNA is increasingly being used as an ideal material for the construction of nanoscale structures, circuits, and machines. Toehold-mediated DNA strand displacement reactions play a very important role in these enzyme-free constructions. In this study, the concept of metallo-toehold was utilized to further develop a mechanism for strand displacement driven by Ag^+^ ions, in which the intercalation of cytosine–cytosine mismatched base pairs on the toeholds provides additional control by varying of the concentration of Ag^+^ ions. The characteristics of displacement reaction in response to different concentration of Ag^+^ ions are investigated by fluorescence spectral and non-denaturing polyacrylamide gel electrophoresis. The reaction can successfully occur when the concentration of Ag^+^ ions is suitabe; excess Ag^+^ ions block the reaction. Furthermore, the displacement reaction can be tuned and controlled most efficiently under the condition of two C:C mismatched base pairs placed on the six-nt toehold. Based on our research, a mechanism was developed to construct Boolean logic gate AND and OR by employing strand displacement reaction as a tool, Ag^+^ and Hg^2+^ as input.

## Introduction

The remarkable specificity and strength of interactions between complementary nucleotides make DNA a useful material [1] for structuring nanoscale device [2]–[4], circuits [5], [6], and machines [7], [8]. Recently, the concept of toehold-mediated DNA strand displace- ment first used by Yurke [9] attracted a lot of interest, which occurs when hybridization of an invading strand starts at a short single strand attached to another single-stranded sticky end called as “toehold” domain of a double-stranded complex, resulting in a branch migration reaction [10] that the invading strand displaces the target strand from the double-stranded complex along with the production of a new complex with the help of a short sequence of contiguous complementary bases. This concept has been proved to be a powerful tool that allows control over the building of nucleic acid tweezers [11], DNA walkers [12], molecular gears [13] as well as the constructing of DNA-based logic gates [14]. Moreover, the Winfree group reported that toehold-mediated strand displacement can be applied to the construction of entropy-driven catalytic circuit reactions [15], digital logic circuit [16], and neural network computation [17]. The kinetics of DNA strand displacement has also been elaborately studied [18]. In previous papers, we also revealed a strategy for the assembly and logic operation of gold nanoparticles driven by a dynamic DNA-fueled molecular machine [19].

Research on interactions between metal ions and nucleic acids has gained increasing widespread attention [20], [21]. When hydrogen-bonded Watson–Crick base pairs are replaced by metal–ligand interactions inside the DNA double helix, a “metallo-base pair” is formed and stabilized through coordination of the ions to the oligonucleotides in certain way [20], [22]–[24]. Certain metal ions can be coordinated by specially designed ligand nucleosides, placed opposite each other in the double helix [25]–[29]. The Ono and Togashi groups have done a lot of innovative work in this area and obtained a series of achievements [30]–[32]. They have reported that T-T and C-C mispairs can specifically capture Hg^2+^ ions and Ag^+^ ions respectively [33]. As metal-base pairs are highly selective and sensitive for specific metal ions, extremely wide range of applications such as DNA sensor for detecting metal ions [34], DNA detection triggered by metal ions [35], construction of DNA nanomachine and nanodevice [36], and fabrication of molecular-scale logic gate [37]–[39], have been widely developed.

Although it has achieved successful applications, the studies of metallo-base pairs have been primarily focused on the sensors with sensitive responses functioned through the interactions between ions and oligonucleotides. Meanwhile, large amount of applications of toehold-mediated DNA strand displacement have almost been focused on pure DNA systems. Controlling the binding strength of the toehold domain can tune the rate of DNA hybridization reaction, but fine adjustment is difficult because the rate varies roughly exponentially with the binding strength [18]. Adjustment is neither convenient through the mechanism of “remote” toeholds presented by the Turberfield group because of the requirement to design many different length DNA to obtain fine result [40].

In a previous paper, we introduced the concept of ‘‘metallo-toeholds’’ in which the toehold domains feature T:T mismatched bases. We demonstrated that the reaction rates can be tuned and controlled dynamically at room temperature through variation of the concentration of Hg^2+^ ions [41]. Recently, displacement reaction triggered by functional toeholds formed such as ions [42] and micromolecule [43], has also been investigated. Herein we further utilize the concept of ‘‘metallo-toeholds’’ in which the toehold domains feature C:C mismatched bases. As expected, this system can be triggered by Ag^+^ ions. We explore the effects of the length of toehold, the numbers of cytosine-cytosine mismatched, and the concentration of Ag^+^ ions on the control of strand displacement reaction. Moreover, a mechanism was further developed for constructing Boolean logic gate AND and OR by employing strand displacement reaction as a tool, Ag^+^ and Hg^2+^ as input.

## Results and Discussion

### Mechanism of Ag^+^–toehold DNA strand displacement

**Figure 1:**
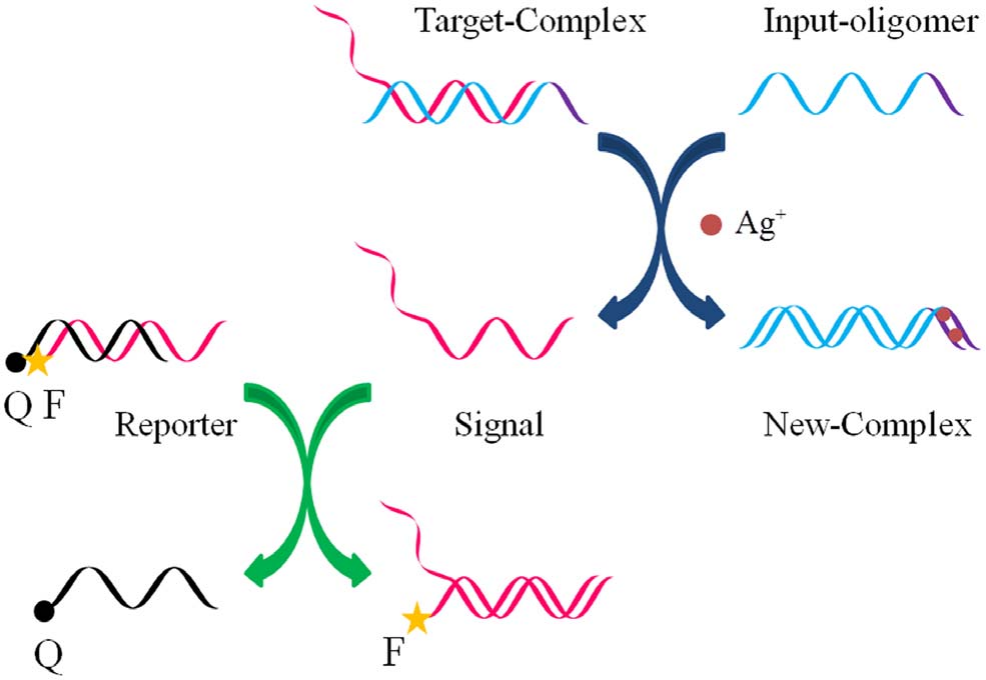
Schematic representation of DNA strand displacement triggered by Ag^+^ ions and fluorosence measurements strategy. F denotes ROX fluorophore, Q denotes BHQ-2 quencher. The reporter complex reacts stoichiometrically with the product Signal to induce an increase in fluorescence intensity.

The software NUPACK [44] was used to carefully design the experimental DNA sequences such that they would possess minimal unwanted secondary structures. All sequences we used are listed in Table S1. As illustrated in Fig. 1, when Ag^+^ is introduced, the Input-oligomer firstly displaces the Signal strand on the Target complex through a toehold-mediated strand displacement, synchronously generating a New complex and releasing the Signal strand. Fluorescence experiments with the use of an additional fluorescence reporter complex was performed. When the input-oligomer and Ag^+^ ions were added to trigger the reaction, the Signal strand undergoes further reaction with the reporter complex, separating the fluorophore from the quencher, thereby inducing an increase in the fluorescence signal. Therefore, the progress of the strand displacement reaction can be observed by measuring the fluorescence intensity. The influence of Ag(I) ions on the fluorophore-labeled oligomer is negligible compared to the quencher (Fig. S1, ESI†).

### Spectrfluorimetry studies

**Figure 2:**
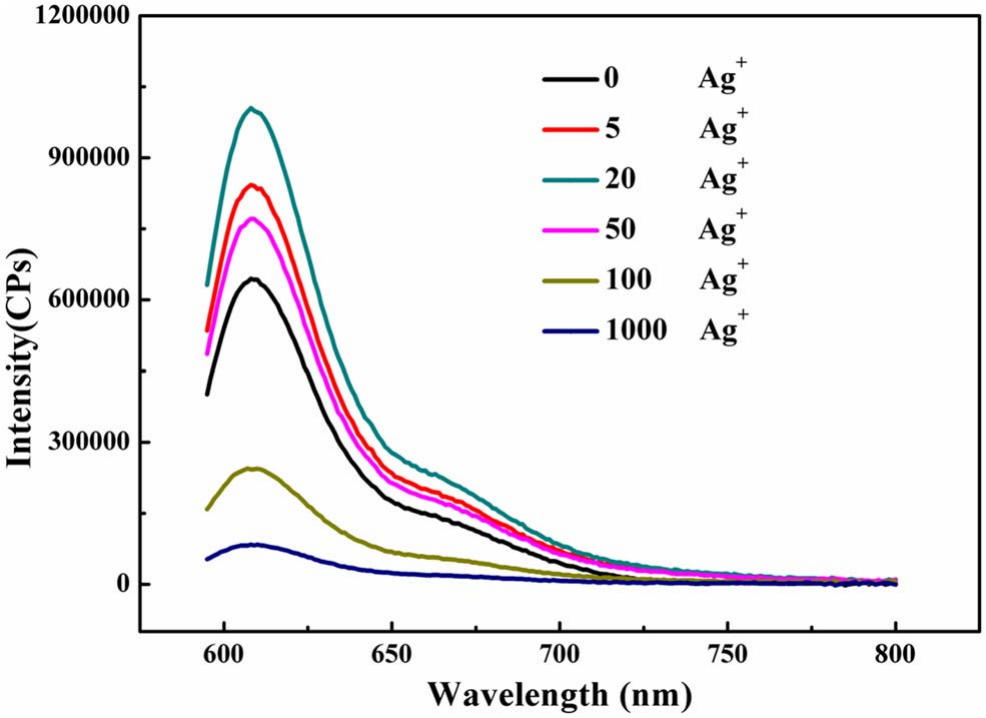
Fluorescence spectra of the reactions after treatment with Ag^+^ ions for 24 h. Final concentration: [Target complex] = 100 nM; [Fluorescence reporter complex] = 150 nM; [Input-oligomer] = 400 nM. The number in the figure represented the ratio [Ag^+^]/[Target complex].

To systematically characterize the status of metallo-toehold DNA strand displacement reaction, typical fluorescence spectral characteristics of the reactions in response to different concentration of Ag^+^ ions after 24 h was performed. First, the experiment in the condition of six-nt toehold with two mismatched site was performed and analyzed. As revealed in Fig. 2, in the absence of Ag^+^ ions, the fluoroscence intensity was relatively low, because two cytosine–cytosine mismatches in the six-nt toeholds between Input-oligoner and Substrate weaken the binding strength, therefore, toehold-mediated DNA strand displacement reaction didn't occur successfully. The fluoroscence intensity increased along with the concentration of Ag^+^ ions increasing to 20×. It is inferred that, in the presence of Ag^+^ ions, Ag^+^ ions inserted into the C:C mismatched base pair to form C–Ag(I)–C structure, similarly to normal base pair, therefore, producing the appearance of a completely complementary toehold, which induced the metallo-toehold-mediated DNA strand displacement to release the Signal strand, which separated the fluorophore from the quencher and, therefore, enhanced the fluorescence signal. The fluorescence signal decreased significantly at higher concentrations of Ag^+^ ions; the transition concentration of Ag^+^ ions was nearly at 50×. When the concentration of Ag^+^ ions was greater than 100×, the fluorescence intensity was even far less than that with the absence of Ag^+^ ions which showed that the displacement reaction was badly blocked. It is supposed that because of special interaction between Ag^+^ ions and cytosine, when the Ag^+^ ions are excessive, the cytosine base is blocked by excess Ag^+^ ions, thereby, the displacement reaction is disrupted. The shapes and intensity of the signals the fluorescence spectra in response to the Ag^+^ ions after 48 h (Fig. S2, ESI†) was similar to those in Fig. 2. Besides, the fluorescence intensity after 48 h was only slightly higher than that after 24 h. In other words, equilibria achieved almost completely within 24 h for the reaction. Thus, a suitable range of concentration of Ag^+^ ions successfully triggered the metallo-toehold-mediated DNA strand displacement reaction. Moreover, the reaction can be tuned through variation of the concentrations of the Ag^+^ ions.

### Native polyacrylamide gel electrophoresis (PAGE)

**Figure 3:**
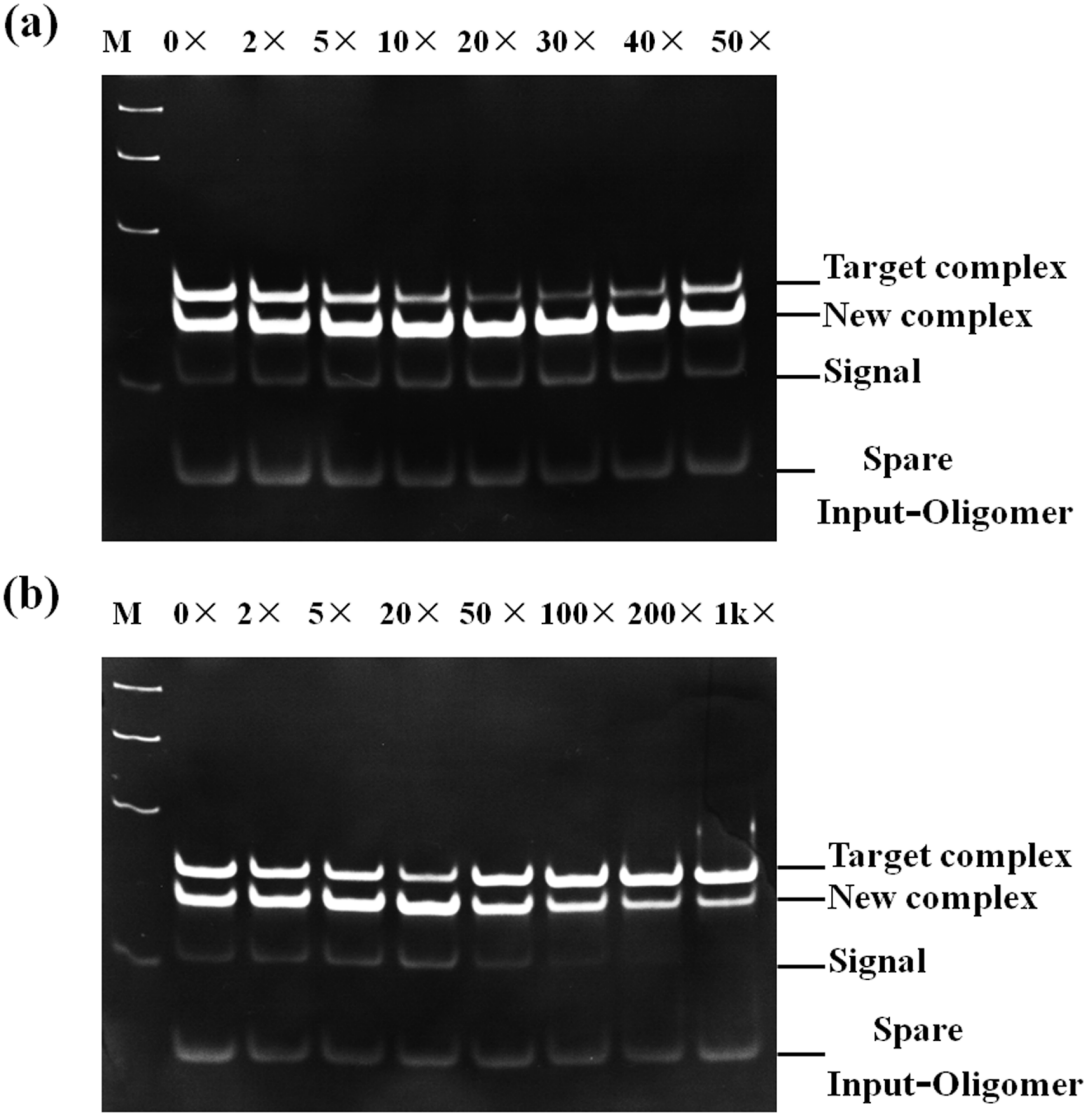
Gel electrophoresis images of the strand displacement reaction triggered by Ag^+^ ions, obtained after 24 h. Lane M: Ladder size markers. The number above each lane represents the relative concentration of Ag^+^ ions to the target complex.

Fig. 3 displays gel electrophoresis images obtained for the strand displacement reaction triggered by a concentration gradient of Ag^+^ ions after 24 h. As expected, a completely complementary metallo-toehold consist of C–Ag(I)–C structure triggered by a suitable concentration range of Ag^+^ ions obviously promoted the strand displacement. Notably, the reaction can be well tuned through regulating the concentration of Ag^+^ ions. Mismatched base pairs on the toehold between Input-oligomer and Substrate induced an obstacle in toehold binding, which played a key role in initiating strand displacement, thereby, slowed down the reaction. The intercalation of Ag^+^ ions made the metallo-toeholds perfect complementary, which drived the strand displacement successfully. Excessive Ag^+^ ions presumably blocked the cytosine sites on the toehold, and then impeded the reaction. Focused on Fig. 3(a), the reaction capacity was increasingly becoming powerful along with the concentration of Ag^+^ ions rising to 40×; however, the reaction started to be impeded at 50×, this was an obvious transition point for the reaction. Moreover, a suitable concentration range of Ag^+^ ions for preferably promoting metallo-toehold DNA strand displacement was about from 10× to 40×. Once the Ag^+^ ions were more than 100-fold excess in Fig. 3(b), the strand displacement reaction was badly hindered. The above discussion reveald that the PAGE data are in good agreement with those from the fluorescence spectra.

### Influences of only one C:C mismatched on the toehold

We further explored the influences of different toehold lengths including five-nt, six-nt, and seven-nt, and the C:C mismatched numbers including one, two. The sequences used in this section are listed in Table S1. To distinguish different DNA strand, we defined the name of DNA as in Table S1.

**Figure 4:**
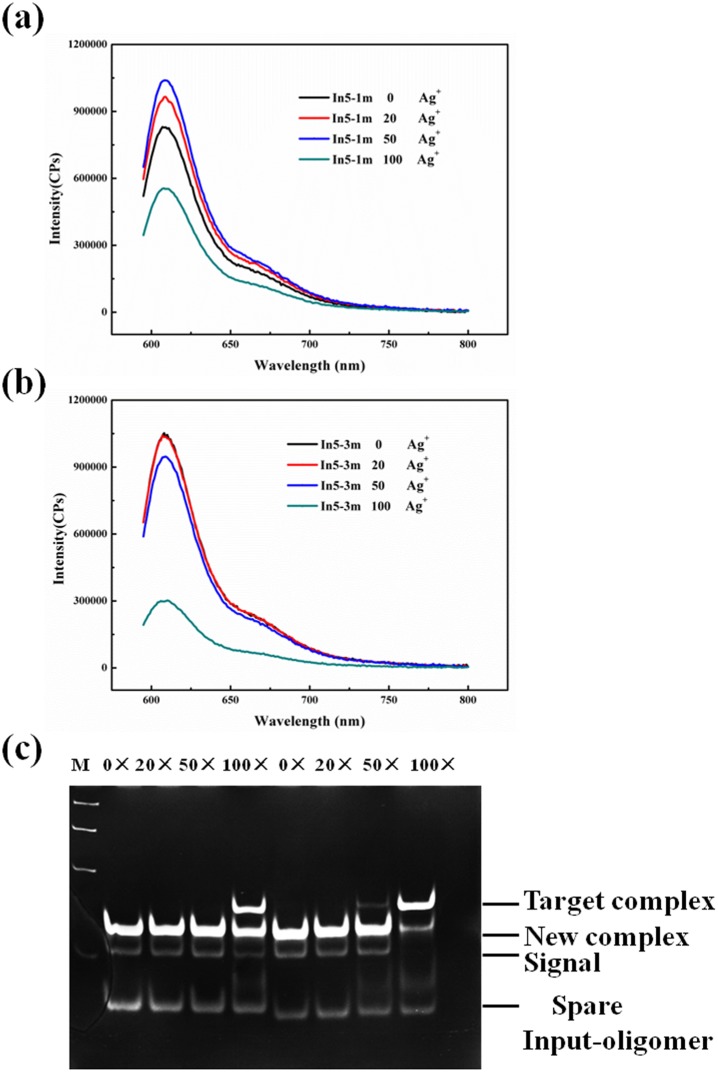
Fluorescence spectra of the reactions after treatment with Ag^+^ ions for 24 h. Final concentration: [target complex] = 100 nM; [fluorescence reporter complex] = 150 nM. Target complex consists of Signal and S5-1,3. (a) [In5-1 m] = 400 nM. (b) [In5-3 m] = 400 nM. The number in the figure represented the ratio [Ag^+^]/[Target complex]. (c) Gel electrophoresis images of strand displacement reaction triggered by Ag^+^ ions, obtained after 24 h. Except for the lane M, that first 4 lanes correspond to In5-1 m and the last four correspond to In5-3 m respectively. The number above each lane represents the relative concentration of Ag^+^ ions to the target complex.

Fluorescence spectral was performed to monitor the characteristic of the reactions in response to the condition of only one C:C mismatched with different length toehold of the Input-oligomer at different concentration of Ag^+^ ions. Fig. 4a and Fig. 4b displays the fluorescence spectral of the reaction that there was only one mismatched on five-nt toehold of In5-1 m and In5-3 m triggered by a concentration gradient of Ag^+^ ions after 24 h. Unexpectedly, the strand displacement reaction completed with five-nt toehold whether the C:C mismatched are placed on either site of the toehold at relative low concentration of Ag^+^ ions, the reaction speeded up slightly along with the increase of Ag^+^ ions; but 100-fold excess Ag^+^ ions obviously impeded the reaction, which was a transition point. The DNA strand displacement reaction rate is positively associated to the length of toehold [18]. Therefore, in the condition of six-nt and seven-nt toehold of Input-oligomer, the reaction more completely occured as expected (Figs. S3 and S4, ESI†); and the transition piont was the same as previously mentioned. To sum up. the strand displacement reaction can not be effectively tuned and controlled under the condition of only one C:C mismatched placed on the toehold which length was from five-nt to seven-nt. Fig. 4c displays gel electrophoresis images of the strand displacement reaction triggered by Ag^+^ ions for only one C:C mismatched on different site at five-nt toehold, obtained after 24 h. As shown, the DNA displacement reaction was close to complete at low concentration of Ag^+^ ions; the reaction appeared to be impeded at 100-fold excess Ag^+^ ions. The PAGE data are in fairly good agreement with those from the fluorescence spectra. Accordingly, we infer that when the concentration of Ag^+^ ions is low, only one mismatched on the toehold does not have reamarkable influence on the binding ability of toehold when the length of toehold is long enough, thus, the strand displacement reaction is neither influenced observably by only one mismatched on the toehold.

### Influences of two C:C mismatched on the toehold

**Figure 5:**
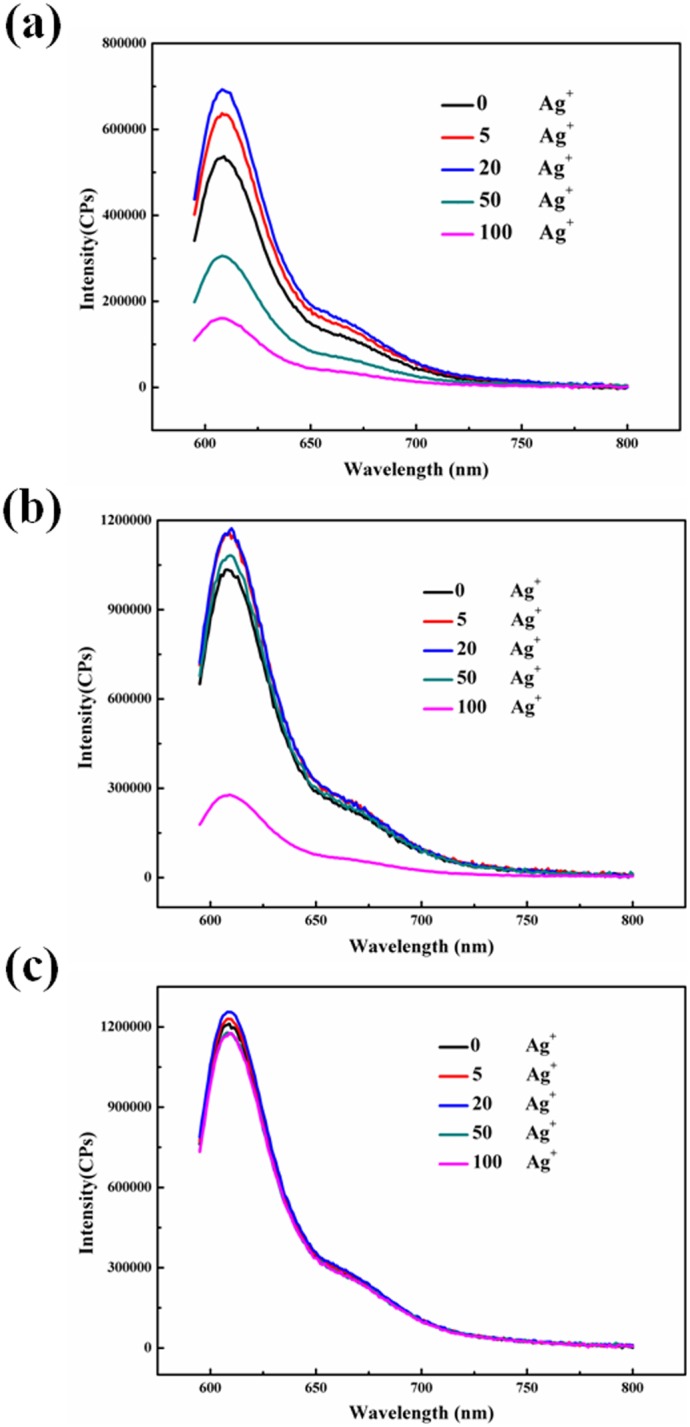
Fluorescence spectra of the reactions after treatment with Ag^+^ ions for 24 h. Final concentration: [target complex] = 100 nM; [fluorescence reporter complex] = 150 nM; [Input-oligomer] = 400 nM. The number in the figure represented the ratio [Ag^+^]/[Target complex]. (a) Target complex is consist of Signal and S5-1, 3; In5-1,3 severs as Input-oligomer. (b) Target complex is consist of Signal and S7-1,3; In7-1,3 severs as Input-oligomer. (c) The completely complementary strand of substrate severs as input-oligomer, all other chains are the same as in Fig. 1.

Fig. 5 displays fluorescence spectral of the reaction that there was two C:C mismatched on five-nt and seven-nt toehold of Input-oligomer triggered by Ag^+^ ions after 24 h. Compared with Fig. 1, the shapes of the signals was similar; besides, the rule of fluorescence signal varied which increased at the beginning and then decreased along with the increasing concentration of Ag^+^ ions was also similar. However, the maximum fluorescence signal at 20× was much less than that in Fig. 1, showing that the binding ability of five-nt toehold with two C:C mismatched was weaker than that of six-nt toehold with two C:C mismatched, inducing that the strand displacement reaction at the equilibrium point did not completely occur at the optimum concentration of Ag^+^ ions. Moreover, the difference of signal intensity between a suitable concentration range of Ag^+^ ions was too small to distinguish clearly. As revealed in Fig. 5(b), the strand diplacement reaction almost entirely occured when the concentration of Ag^+^ ions was less than 50×; as expected, the reaction was prevented when the concentration of Ag^+^ ions was more than 10 µM, which is equal to 100-fold excess. In conclusion, the strand displacement reaction can not be tuned and controlled efficiently under the condition of two C:C mismatched placed on the five-nt toehold and seven-nt toehold. To verify the effect of excess Ag^+^ ions, fluorescence spectral for introducing completely complementary strand of Substrate severed as Input-oligomer was also performed in Fig. 5(c). Visibly, normal DNA strand displacement reaction almost unaffected by Ag^+^ ions which shows that excessive Ag^+^ ions almost have no influences on the formation of normal base-pair. Hereby, the conclusion can be obtained that when Ag^+^ ions is extremely excessive, the cytosine base will be surrounded even wrapped by excess Ag^+^ ions, thus, the formation of C–Ag(I)–C structure will be blocked, thus, the DNA strand displacement reaction will also be predictably prevented.

### Development of “AND” and “OR” logic gates

According to our research and the influences of the toehold length on DNA displacement [45], a simple and universal logic system were developed where Ag^+^ and Hg^2+^ ions worked as the input and fluorescence intensity worked as the output. Both one C:C and one T:T mismatch base pair, which can be specifically inserted by Ag^+^ and Hg^2+^ ions respectively, were introduced on the toehold for the design of logic operation. The inputs are Ag^+^ and Hg^2+^, with the absence and presence of each ion defined as “0” and “1”, respectively. To more intuitively express the output, a value (F–F_0_)/F_0_ (F0 and F represent the fluorescence intensity of the system in the absence and presence of metal ions, respectively) was employed as output, if the value is higher than 1, the output is defined as “1”, and the others were defined as “0”.

**Figure 6:**
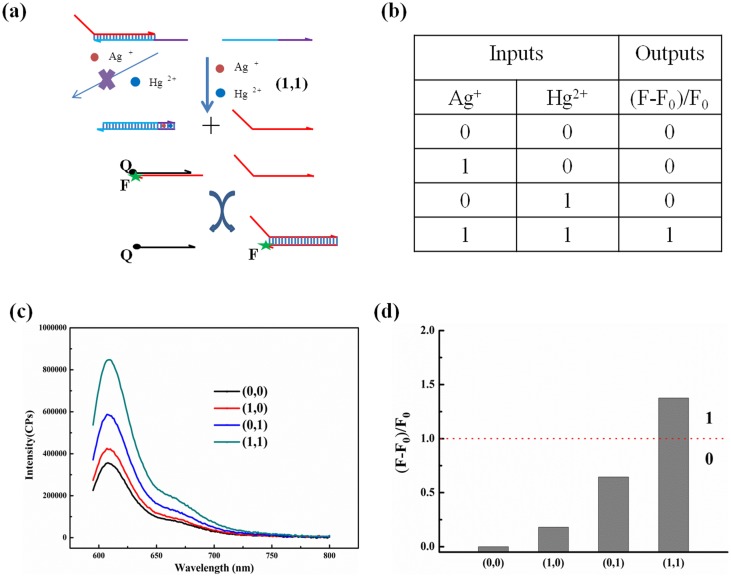
The ‘AND’ logic gate system using Hg^2+^ and Ag^+^ as inputs while the enhancement of fluorescence intensity as output. (a) The design of the logic gate. (b) The truth table of the logic gate. (c) The resultant fluorescence spectra of different input states. (d) Values of (F–F_0_)/F_0_ respond to different input states.

Fig. 6a reveals the design of “AND” logic gate with metal ions inputs. In the absence of either or both inputs, there are at least one mismatch base pairs placed on five-nt toehold, weakening the binding strength of toehold, so DNA displacement will be impeded. Only in the presence of both inputs, the toehold is completely complementary in appearance because Ag^+^ ions and Hg^2+^ ions can insert into and stabilize C–C and T–T mismatch base pairs, thereby, the DNA displacement can successfully occur, leading to increase the fluorescence intensity. A truth table is shown in Fig. 6b. Fig. 6c reveals the fluorescence intensity of “AND” logic gate with metal ions inputs. In the absence of both Ag^+^ and Hg^2+^ inputs (0,0), or in the presence of either Ag^+^ (1,0), or Hg^2+^ input (0,1), the fluorescence intensity was relatively weak. Only in the presence of both Ag^+^ and Hg^2+^ (1,1) caused great increase of fluorescence intensity. The value of (F–F_0_)/F_0_ were calculated in Fig. 6d. It shows that only under the input (1,1) the value was higher than 1, which corresponded to the true output; the value of other inputs were all smaller than 1, which corresponded to the false output. PAGE gel electrophoresis was performed to qualitatively test the design of “AND” logic gate (Fig. S5). Obviously, only in the presence of both inputs (1,1), the Signal band was clearly visible which shows that the DNA displacement occurred successfully, that is to say, only the (1,1) input obtained a output 1, other inputs obtained a output 0. The PAGE result is in good agreement with that from the fluorescence spectra.

**Figure 7:**
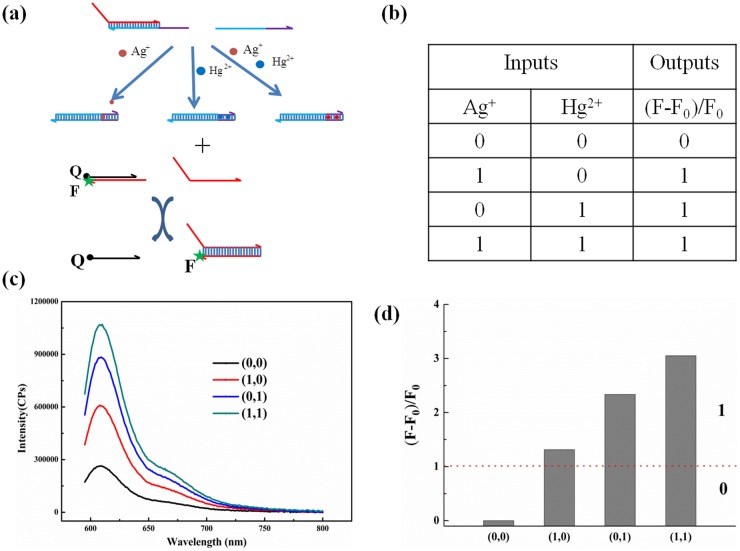
The ‘AND’ logic gate system using Hg^2+^ and Ag^+^ as inputs while the enhancement of fluorescence intensity as output. (a) The design of the logic gate. (b) The truth table of the logic gate. (c) The resultant fluorescence spectra of different input states. (d) Values of (F–F_0_)/F_0_ respond to different input states.

Fig. 7a reveals the design of “OR” logic gate with metal ions inputs. In the absence of both Ag^+^ and Hg^2+^ inputs, there are two mismatch base pairs placed on six-nt toehold, which means that the toehold binding strength was weak, thereby, the DNA displacement will be blocked. In the presence of either or both input, one of two mismatch base pairs can be stabilized by the formation of C–Ag–C or T–Hg–T, so the binding ability between toeholds are strong enough to trigger the DNA displacement, thereby, inducing the enhancement of fluorescence intensity. A truth table is shown in Fig. 7b. Fig. 7c reveals the fluorescence intensity of “OR” logic gate with metal ions inputs. In the absence of both Ag^+^ and Hg^2+^ inputs (0,0), the fluorescence intensity was much weaker than others; while in the presence of either Ag^+^ (1,0), or Hg^2+^ (0,1) input, or both inputs (1,1), the fluorescence intensity increased greatly. The value of (F–F_0_)/F_0_ were calculated in Fig. 7d. It can obviously seen that only the absence of both inputs obtained a false output, while the presence of either input or both inputs obtained a true output. The fluorescence intensity of (1,0) input was visibly smaller than that of (0,1) input because that the binding affinity of C–Ag–C is weaker than that of T–Hg–T [21]. PAGE gel electrophoresis was also performed to qualitatively test the design of “OR” logic gate (Fig. S6). Except for the (0,0) input, the Signal band was clearly visible in all other inputs which shows that the DNA displacement occurred successfully, that is to say, only the (0,0) input obtained a output 0, other inputs obtained a output 1. The PAGE result is in good agreement with that from the fluorescence spectra.

## Conclusions

In summary, the concept of metallo-toeholds is utilized to further propose a mechanism that Ag^+^ ions which specifically insert into mismatched base pair (C:C) to form C–Ag(I)–C structure can be used to trigger the DNA strand displacement reaction. The Ag^+^ ions serve as a regulator, and can tune the DNA strand displacement reaction through metallo-toehold. Through detailed discussions on the influences of toehold length and C:C mismatched numbers on the toehold, we confirmed that the strand displacement reaction can be tuned and controlled most efficiently under the condition of two C:C mismatched placed on a six-nt toehold. By introducing the completely complementary strand, we clarified that excessive Ag^+^ ions obstructed the dis- placement reaction. Moreover, a mechanism for constructing Boolean logic gate AND and OR by employing strand displacement reaction as a tool, Ag^+^ and Hg^2+^ as input is deveploed. Besides, such logic gates work well. It has been reported that other metal ions, such as Cu^2+^ and Ni^2+^, can specifically interact with nucleoside bases to form metal-ion-mediated base pairs [46], [47]. Therefore, our strategy extends the application between metal ions and nucleic acids. Moreover, this strategy can be further used to construct new logic gate with other metal ions as input and complicated logic circuits. We hope that the concept of metallo-toehold formed by metal ions (e.g., Ag^+^ and Hg^2+^) can be further used in DNA nanotechnology.

## Materials and Methods

### Materials

All DNA oligomer sequences used in this study were synthesized by Shanghai Sangon Biotechnology (Shanghai, China), all non-modification oligomer purified by UltraPAGE, the modification oligomer of fluorophores and quencher purified by HPLC. The DNA sequences tested in Scheme 1 are presented in Table S1. All other sequence are presented in Table S1. Tris(hydroxymethyl) aminomethane (Tris), anhydrous sodium acetate (NaOAc), magnesium acetate tetrahydrate [Mg(OAc)_ 2_], potassium nitrate (KNO_3_), silver(I) nitrate (AgNO_3_) were purchased from Sinapharm Chemical Reagent (Shanghai, China). Mercury(II) nitrate (Hg(NO_3_)_2_) was obtained from Wanshan Mineral Products Company (Tongren, Guizhou, China). All salts were of analytical grade and used without further purification. The oligomer samples were dissolved in a buffer consisting of 50 mM Tris-HOAc, 200 mM NaOAc, 0.005 M Mg(OAc)_2_, and 0.03 M KNO_3_ at pH 7.4 [48]. The concentrations of the oligonucleotide solutions were quantified by measuring the UV absorbance at 260 nm. Unless otherwise stated, all other reagents were of analytical reagent grade and used without further purification or treatment. Ultrapure water (Milli-Q plus, Millipore, Bedford, MA) was used throughout. The DNA complex required prior annealing treatment. All annealing processes were performed using a dry bath (ABSON, USA). The samples were heated at 95°C, equilibrated for 15 min at this temperature, and then slowly cooled to 20°C at a constant rate over the course of 90 min.

### Native polyacrylamide gel electrophoresis (PAGE)

Non-denaturing PAGE was run on 20% acrylamide (19∶1 acrylamide:bis), diluted from 40% acrylamide stock purchased from Shanghai Sangon Biotechnology (Shanghai, China); 6× glycerol was used as the loading buffer, was added in 0.2× stochiometry to all samples. Gels were run at 100 V for 200 min at 25°C. Gels were stained 15 min with GelRed, purchased from Biotium (catalog number 41003), then scanned with a Gel Image System (Tanon 1600).

### Spectrofluorimetry studies

Spectrofluorimetry studies were carried out with a commercial SPEX Fluorolog-3 (Jobin Yvon S.A.S, France). The excitation of ROX fluorophre was at 588 nm, and emission was at 608 nm. In all spectrofluorimetry experiments, the total reaction volume was 200 µL. Both the excitation and emission slit widths were set to 2.5 nm, with an integration time of 0.5 second for obtaining smooth curves. Prior to each experiment, all cuvettes were cleaned thoroughly: each cuvette was washed ten times in ultrapure water, another three times in absolute ethanol, and then was flushed with a constant stream of dry nitrogen gas to avoid water condensation.

## Supporting Information

Figure S1 Fluorescence spectra of fluorophore-labeled oligomer in the presence of Ag(I) ions and the quencher-labeled oligomer. Initial concentrations: [fluorophore-labeled oligomer] = 100 nM. The number in the figure represented the ratio [Ag^+^]/[fluorophore-labeled oligomer].
(TIF)

Figure S2 Fluorescence spectra of the reactions after treatment with Ag^+^ ions for 48 h. Final concentration: [Target complex] = 100 nM; [Fluorescence reporter complex] = 150 nM; [Input-oligomer] = 400 nM. The number in the figure represented the ratio [Ag^+^]/[Target complex].
(TIF)

Figure S3 Fluorescence spectra of the reactions after treatment with Ag^+^ ions for 24 h. Final concentration: [Target complex] = 100 nM; [Fluorescence reporter complex] = 150 nM. Target complex consists of Signal and S6-1,3. (a) [In6-1 m] = 400 nM (b) [In6-3 m] = 400 nM. The number in the figure represented the ratio [Ag^+^]/[Target complex]. (c) Gel electrophoresis images of the strand displacement reaction triggered by Ag^+^ ions, obtained after 24 h. Except for the lane M, that first 4 lanes correspond to In6-1 m and the last four correspond to In6-3 m respectively. The number above each lane represents the relative concentration of Ag^+^ ions to the target complex.
(TIF)

Figure S4 Fluorescence spectra of the reactions after treatment with Ag^+^ ions for 24 h. Final concentration: [Target complex] = 100 nM; [Fluorescence reporter complex] = 150 nM. Target complex consists of Signal and S7-1,3. (a) [In7-1 m] = 400 nM (b) [In7-3 m] = 400 nM. The number in the figure represented the ratio [Ag^+^]/[Target complex]. (c) Gel electrophoresis images of the strand displacement reaction triggered by Ag^+^ ions, obtained after 24 h. Except for the lane M, that first 4 lanes correspond to In7-1 m and the last four correspond to In7-3 m respectively. The number above each lane represents the relative concentration of Ag^+^ ions to the target complex.
(TIF)

Figure S5 Gel electrophoresis images of “AND” logic gate. Lane M: Ladder size markers, Lane T: Target complex, Lane S: Signal. (0,0) (1,0) (0,1) (1,1) represent different inputs corresponding to no ions input, Ag^+^ ions input, Hg^2+^ ions input and both Ag^+^ and Hg^2+^ ions inputs, respectively.
(TIF)

Figure S6 Gel electrophoresis images of “OR” logic gate. Lane M: Ladder size markers, Lane T: Target complex, Lane S: Signal. (0,0) (1,0) (0,1) (1,1) represent different inputs corresponding to no ions input, Ag^+^ ions input, Hg^2+^ ions input and both Ag^+^ and Hg^2+^ ions inputs, respectively.
(TIF)

Table S1 DNA sequence tested in the schematic. Bold domain sequences represent the metallo-toehold. Bases in italics formed C:C mismatched base pairs that allowed insertion of a Ag(I) ion.
(DOCX)
