# Supplementary figures and images for: DNA Logic Gate Based on Metallo-Toehold Strand Displacement
Source: PLoS One. 2014 Nov 3;9(11):e111650. doi: 10.1371/journal.pone.0111650 (PMC4218789; doi:10.1371/journal.pone.0111650)

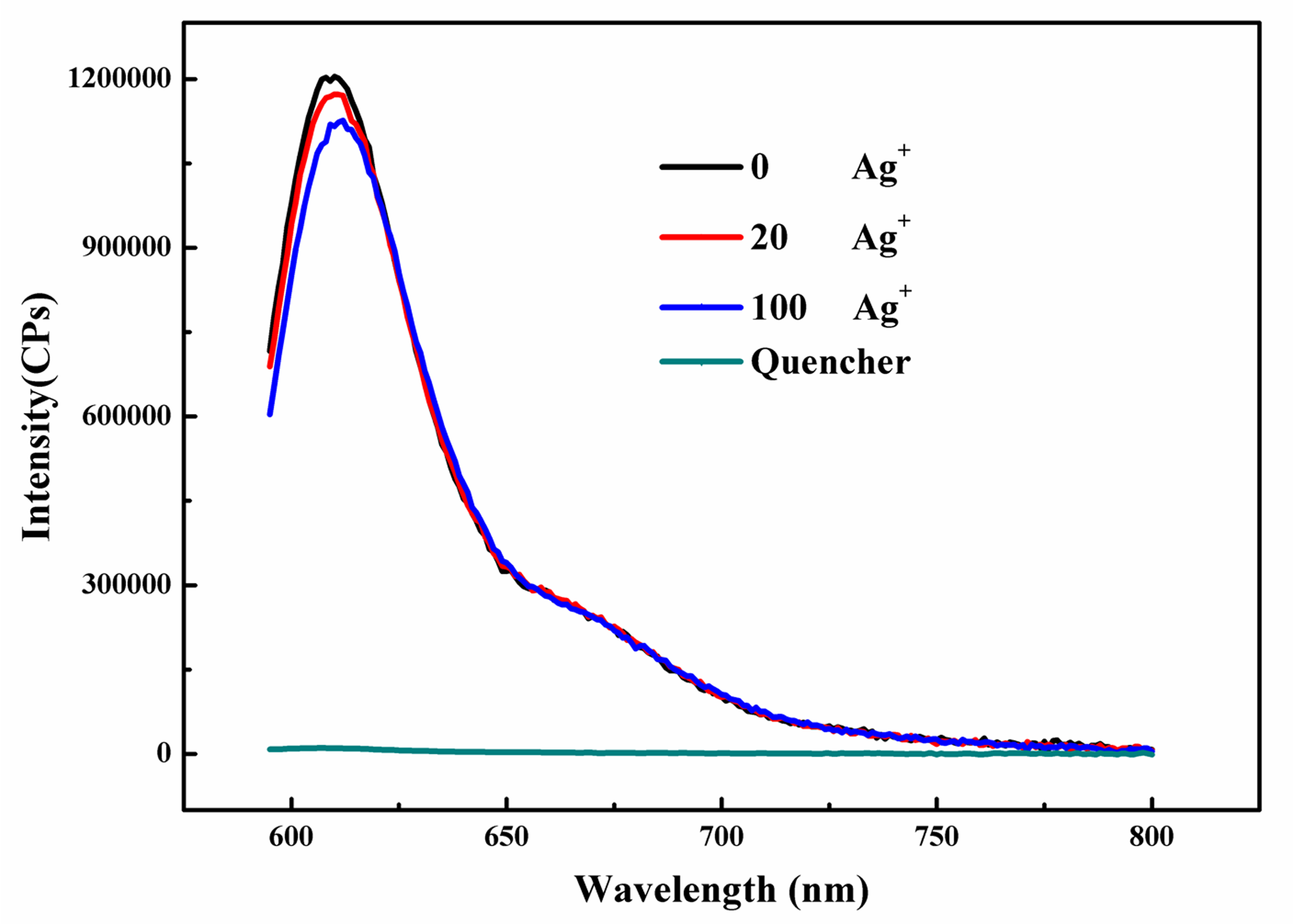

Supplement: Figure S1 — Fluorescence spectra of fluorophore-labeled oligomer in the presence of Ag(I) ions and the quencher-labeled oligomer. Initial concentrations: [fluorophore-labeled oligomer] = 100 nM. The number in the figure represented the ratio [Ag+]/[fluorophore-labeled oligomer]. (TIF) [file pone.0111650.s001.tif]

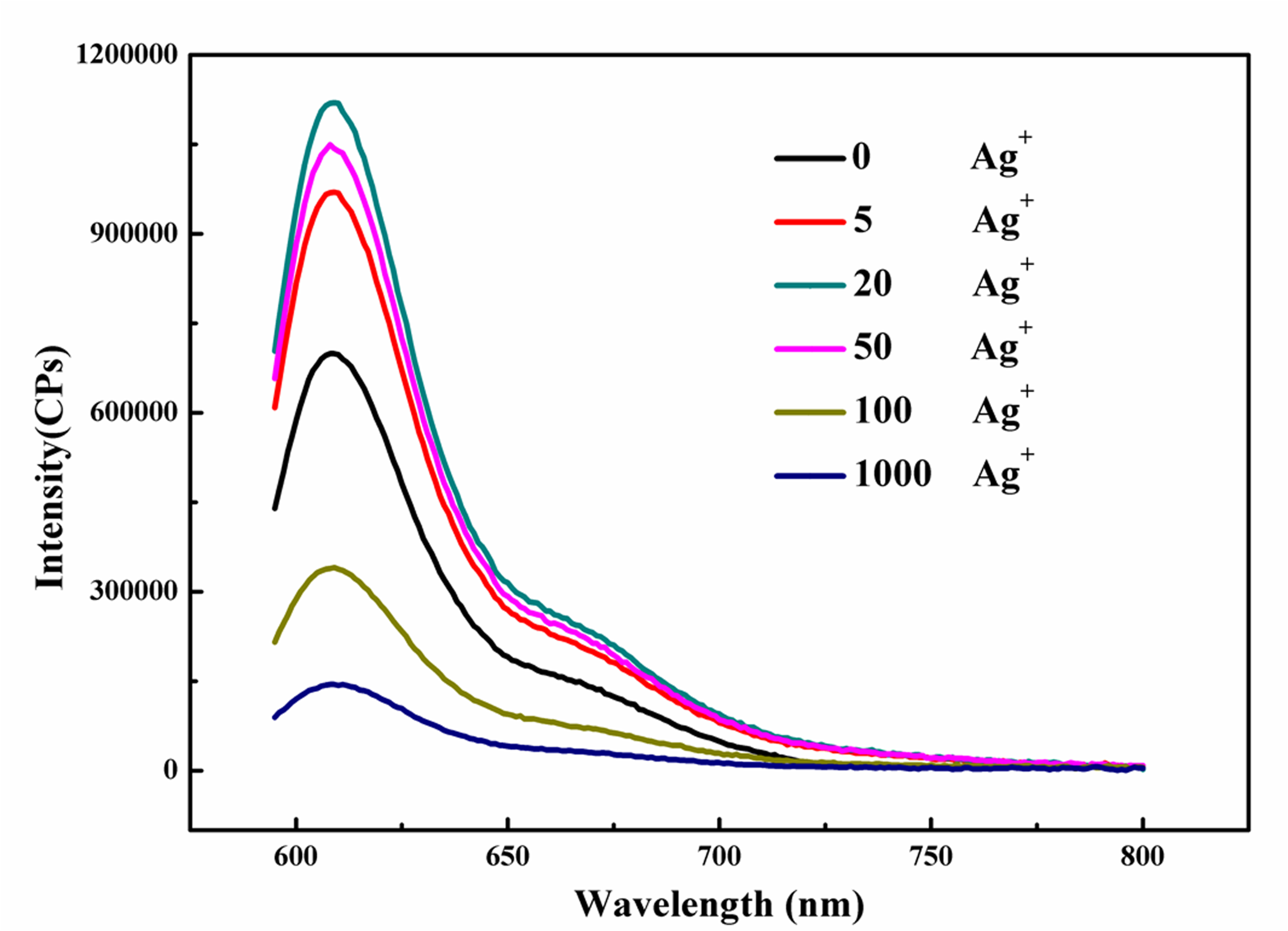

Supplement: Figure S2 — Fluorescence spectra of the reactions after treatment with Ag+ ions for 48 h. Final concentration: [Target complex] = 100 nM; [Fluorescence reporter complex] = 150 nM; [Input-oligomer] = 400 nM. The number in the figure represented the ratio [Ag+]/[Target complex]. (TIF) [file pone.0111650.s002.tif]

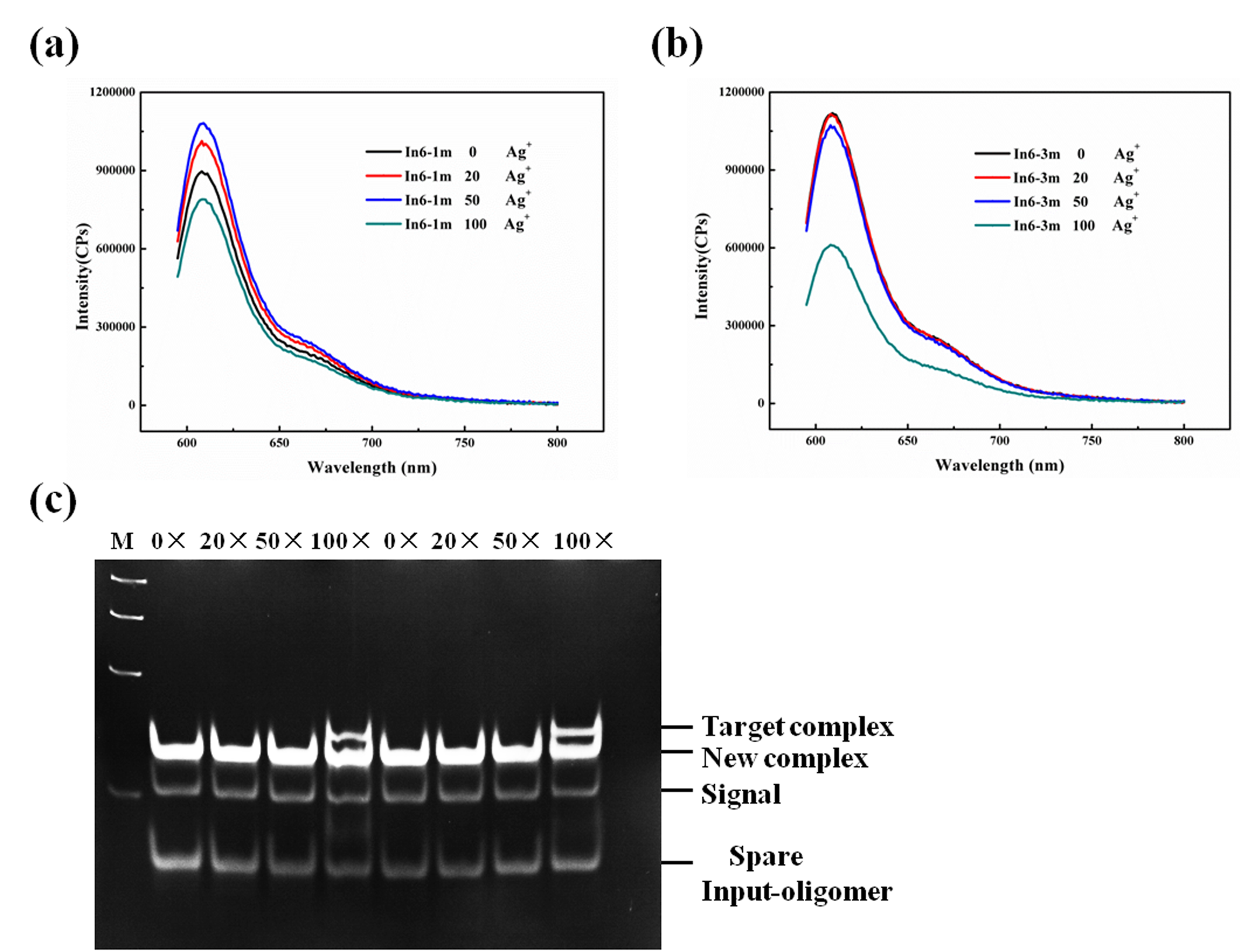

Supplement: Figure S3 — Fluorescence spectra of the reactions after treatment with Ag+ ions for 24 h. Final concentration: [Target complex] = 100 nM; [Fluorescence reporter complex] = 150 nM. Target complex consists of Signal and S6-1,3. (a) [In6-1 m] = 400 nM (b) [In6-3 m] = 400 nM. The number in the figure represented the ratio [Ag+]/[Target complex]. (c) Gel electrophoresis images of the strand displacement reaction triggered by Ag+ ions, obtained after 24 h. Except for the lane M, that first 4 lanes correspond to In6-1 m and the last four correspond to In6-3 m respectively. The number above each lane represents the relative concentration of Ag+ ions to the target complex. (TIF) [file pone.0111650.s003.tif]

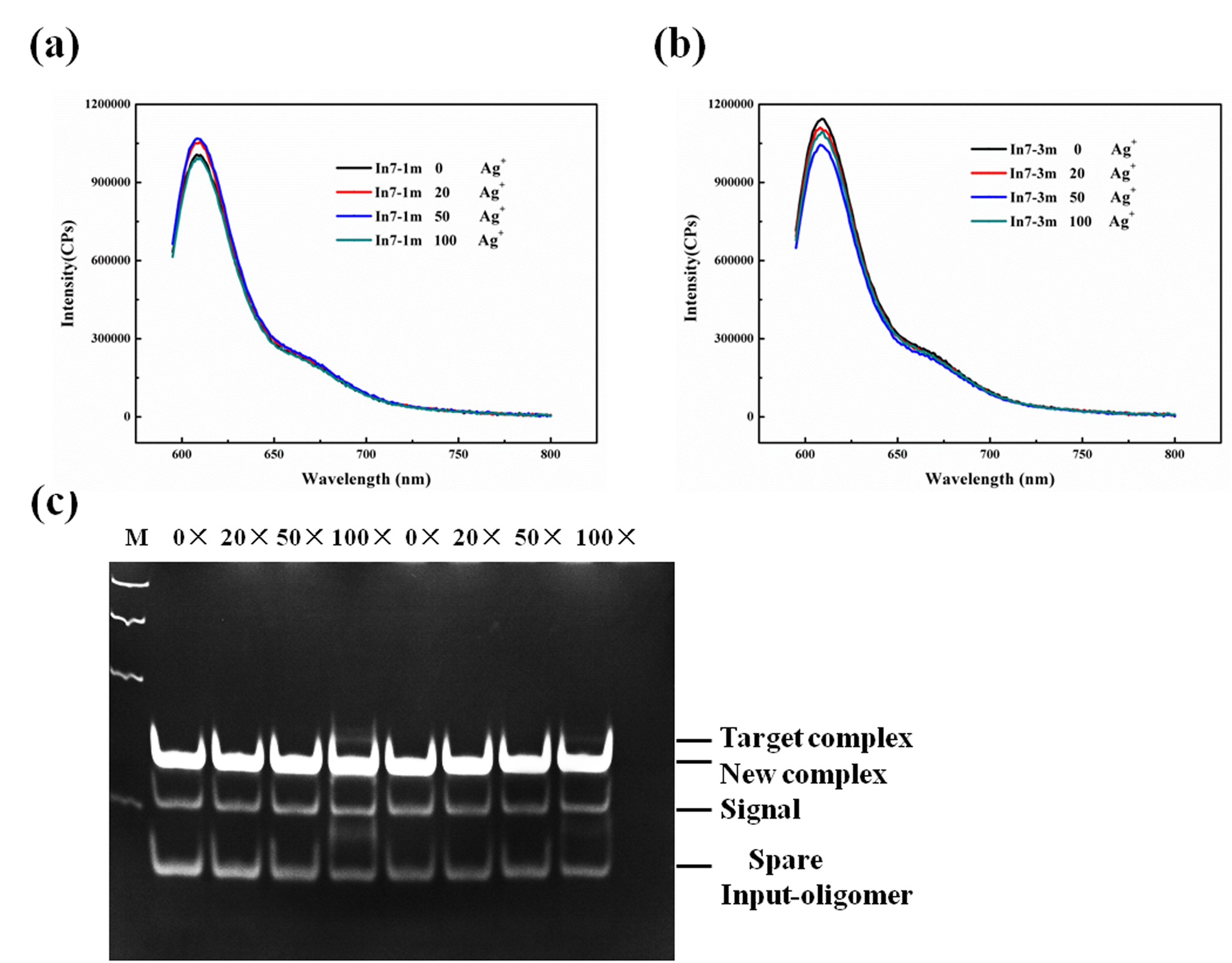

Supplement: Figure S4 — Fluorescence spectra of the reactions after treatment with Ag+ ions for 24 h. Final concentration: [Target complex] = 100 nM; [Fluorescence reporter complex] = 150 nM. Target complex consists of Signal and S7-1,3. (a) [In7-1 m] = 400 nM (b) [In7-3 m] = 400 nM. The number in the figure represented the ratio [Ag+]/[Target complex]. (c) Gel electrophoresis images of the strand displacement reaction triggered by Ag+ ions, obtained after 24 h. Except for the lane M, that first 4 lanes correspond to In7-1 m and the last four correspond to In7-3 m respectively. The number above each lane represents the relative concentration of Ag+ ions to the target complex. (TIF) [file pone.0111650.s004.tif]

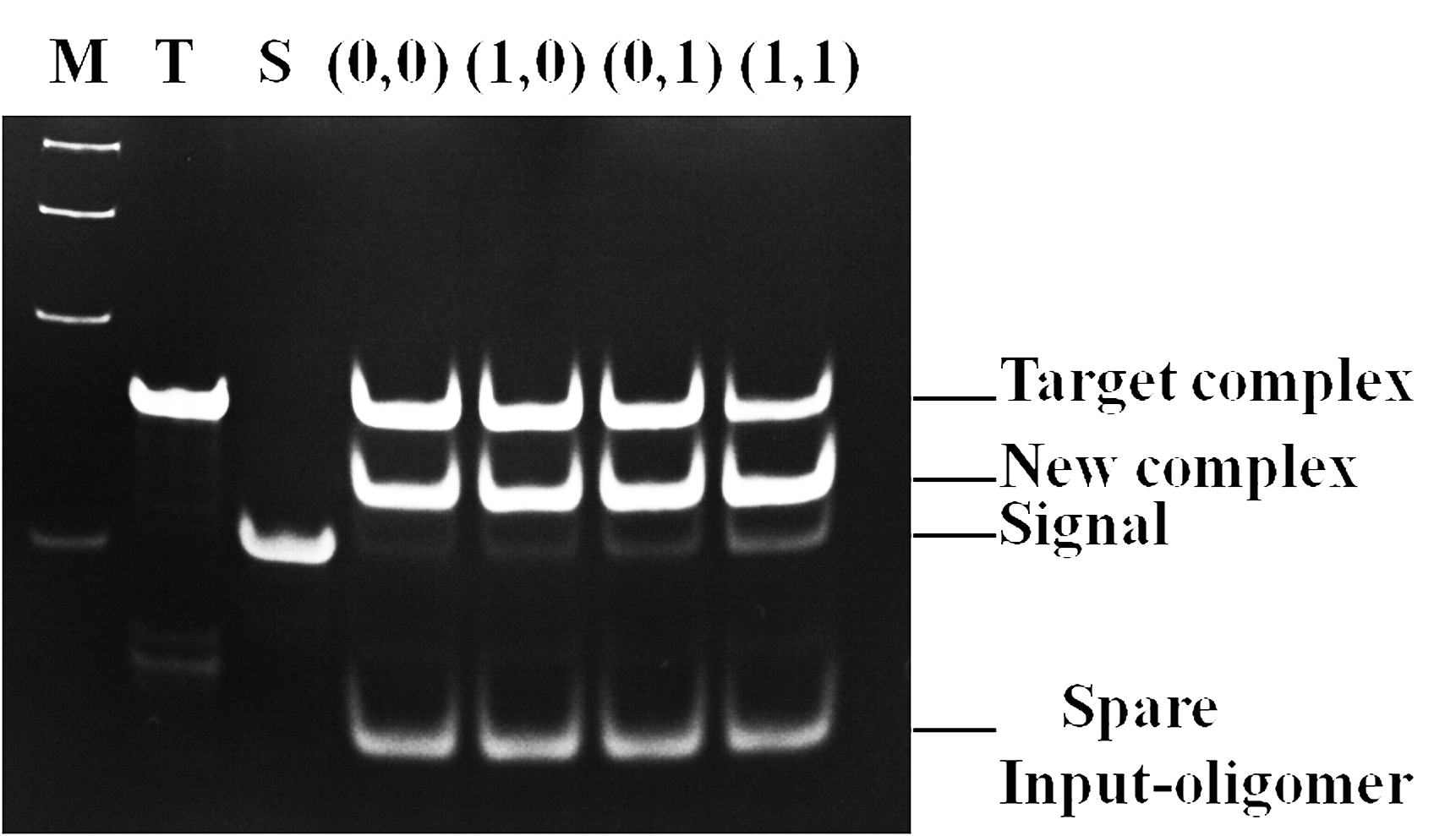

Supplement: Figure S5 — Gel electrophoresis images of “AND” logic gate. Lane M: Ladder size markers, Lane T: Target complex, Lane S: Signal. (0,0) (1,0) (0,1) (1,1) represent different inputs corresponding to no ions input, Ag+ ions input, Hg2+ ions input and both Ag+ and Hg2+ ions inputs, respectively. (TIF) [file pone.0111650.s005.tif]

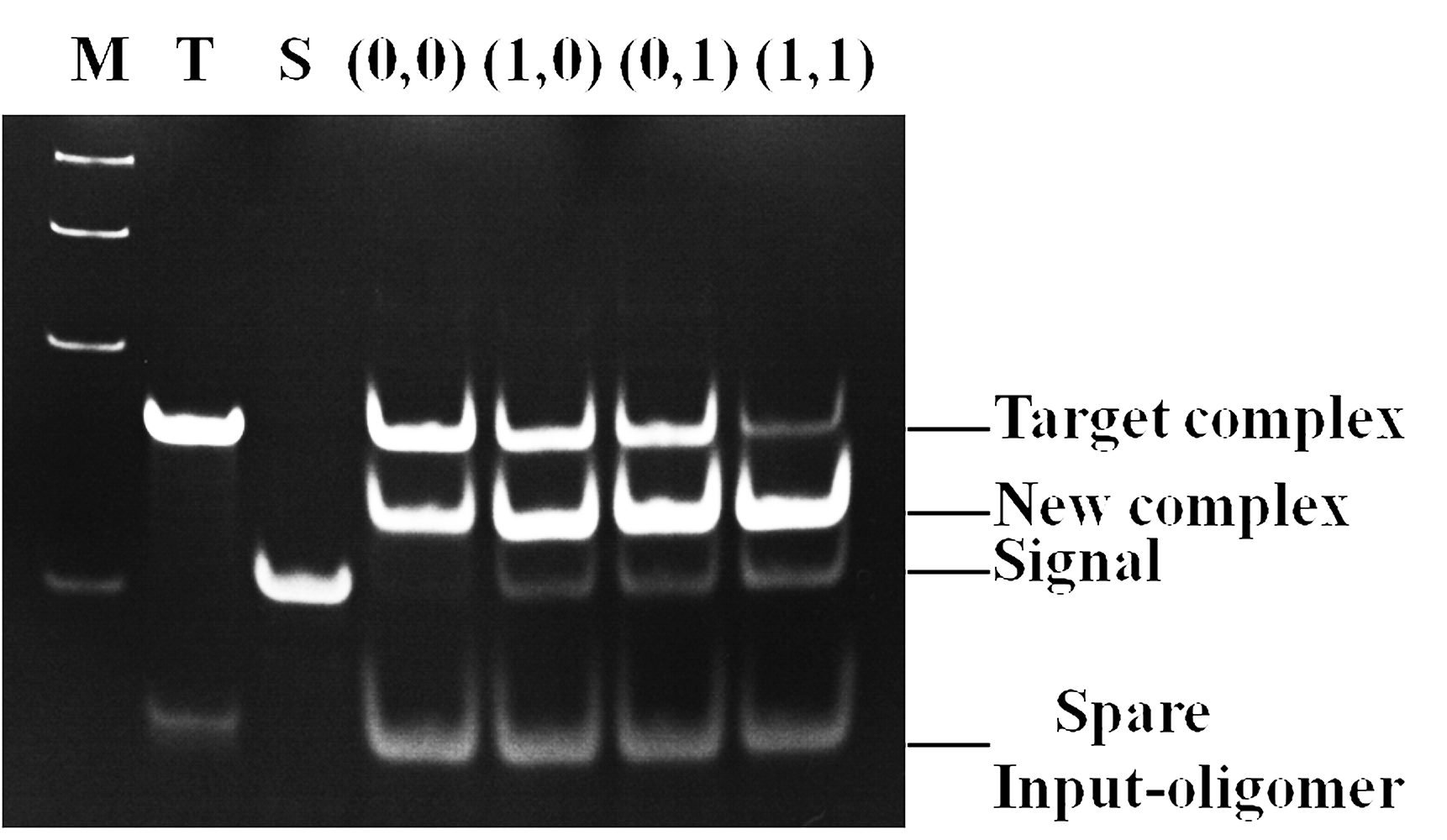

Supplement: Figure S6 — Gel electrophoresis images of “OR” logic gate. Lane M: Ladder size markers, Lane T: Target complex, Lane S: Signal. (0,0) (1,0) (0,1) (1,1) represent different inputs corresponding to no ions input, Ag+ ions input, Hg2+ ions input and both Ag+ and Hg2+ ions inputs, respectively. (TIF) [file pone.0111650.s006.tif]
