# Supplementary material for: DNA Logic Gate Based on Metallo-Toehold Strand Displacement
Source: PLoS One. 2014 Nov 3;9(11):e111650. doi: 10.1371/journal.pone.0111650 (PMC4218789; doi:10.1371/journal.pone.0111650)
Supplement: Table S1 — DNA sequence tested in the schematic. Bold domain sequences represent the metallo-toehold. Bases in italics formed C:C mismatched base pairs that allowed insertion of a Ag(I) ion. (DOCX) [file pone.0111650.s007.docx]

**Table S1** . DNA sequence tested in the schematic. Bold domain sequences represent the metallo-toehold. Bases in italics formed C:C mismatched base pairs that allowed insertion of a Ag(I) ion.

| Name | Complete sequence |
| --- | --- |
| Signal | 5’-CCACATACATCATATTCCCTCATTCAATACCCTACG-3’ |
| Substrate(S) | 5’-GCTCGCCGTAGGGTATTGAATGAGGG-3’ |
| Input-oligomer(In) | 5’-CCCTCATTCAATACCCTACGCCCAGC-3’ |
| Fluorophore-labeled  Oligomer of reporter | 5’-TATTGAATGAGGGAATATGATGTATGTGG-(ROX)-3’ |
| Quencher-labeled  oligomer of reporter | 5’-(BHQ-2)-CCACATACATCATATTCCCTCA-3’ |
| S5-1,3 | 5’-CTCGCCGTAGGGTATTGAATGAGGG-3’ |
| In5-1,3m | 5’-CCCTCATTCAATACCCTACGCCCAG-3’ |
| In5-1m | 5’-CCCTCATTCAATACCCTACGCCGAG-3’ |
| In5-3m | 5’-CCCTCATTCAATACCCTACGGCCAG-3’ |
| Completely complementary of substrate | 5’-CCCTCATTCAATACCCTACGGCGAGC-3’ |
| In6-1m | 5’-CCCTCATTCAATACCCTACGCCGAGC-3’ |
| In6-3m | 5’-CCCTCATTCAATACCCTACGGCCAGC-3’ |
| S7-1,3 | 5’-AGCTCGCCGTAGGGTATTGAATGAGGG-3’ |
| In7-1,3m | 5’-CCCTCATTCAATACCCTACGCCCAGCT-3’ |
| In7-1m | 5’-CCCTCATTCAATACCCTACGCCGAGCT-3’ |
| In7-3m | 5’-CCCTCATTCAATACCCTACGGCCAGCT-3’ |
| AND-Substrate | 5’-TTCCGCGTAGGGTATTGAATGAGGG-3’ |
| AND-Input | 5’-CTCATTCAATACCCTACGCCGTA-3’ |
| OR-Substrate | 5’-GATTGCCGTAGGGTATTGAATGAGGG-3’ |
| OR-Input | 5’-CTCATTCAATACCCTACGCCATTC-3’ |
